# Robust Visual Odometry Leveraging Mixture of Manhattan Frames in Indoor Environments

**DOI:** 10.3390/s22228644

**Published:** 2022-11-09

**Authors:** Huayu Yuan, Chengfeng Wu, Zhongliang Deng, Jiahui Yin

**Affiliations:** 1 School of Electronic Engineering, Beijing University of Posts and Telecommunications, Beijing 100876, China; 2 Science and Technology on Complex System Control and Intelligent Agent Cooperation Laboratory, Beijing 100074, China

**Keywords:** SLAM, localization, mapping

## Abstract

We propose a robust RGB-Depth (RGB-D) Visual Odometry (VO) system to improve the localization performance of indoor scenes by using geometric features, including point and line features. Previous VO/Simultaneous Localization and Mapping (SLAM) algorithms estimate the low-drift camera poses with the Manhattan World (MW)/Atlanta World (AW) assumption, which limits the applications of such systems. In this paper, we divide the indoor environments into two different scenes: MW and non-MW scenes. The Manhattan scenes are modeled as a Mixture of Manhattan Frames, in which each Manhattan Frame in itself defines a Manhattan World of a specific orientation. Moreover, we provide a method to detect Manhattan Frames (MFs) using the dominant directions extracted from the parallel lines. Our approach is designed with lower computational complexity than existing techniques using planes to detect Manhattan Frame (MF). For MW scenes, we separately estimate rotational and translational motion. A novel method is proposed to estimate the drift-free rotation using MF observations, unit direction vectors of lines, and surface normal vectors. Then, the translation part is recovered from point-line tracking. In non-MW scenes, the tracked and matched dominant directions are combined with the point and line features to estimate the full 6 degree of freedom (DoF) camera poses. Additionally, we exploit the rotation constraints generated from the multi-view dominant directions observations. The constraints are combined with the reprojection errors of points and lines to refine the camera pose through local map bundle adjustment. Evaluations on both synthesized and real-world datasets demonstrate that our approach outperforms state-of-the-art methods. On synthesized datasets, average localization accuracy is 1.5 cm, which is equivalent to state-of-the-art methods. On real-world datasets, the average localization accuracy is 1.7 cm, which outperforms the state-of-the-art methods by 43%. Our time consumption is reduced by 36%.

## 1. Introduction

Visual simultaneous localization and mapping (Visual SLAM) and Visual Odometry (VO) estimate the 6 DoF camera pose from a sequence of camera images. They have various applications, such as autonomous robots and virtual and augmented reality (VR/AR).

Indoor environments contain low-texture surfaces such as the floor, walls, and ceiling, which leads to performance degradation for pure point-based methods [1]. Robust pose estimation performance can be improved by adding geometric structural features present in indoor scenes, such as lines and planes, to the systems [2,3,4,5,6,7]. These works extend the working scenarios to low-textured environments.

A technique to leverage the structural regularity in indoor scenes is based on the MW/AW assumption, which can reduce the rotation drift. This technique has been employed by [8,9,10,11,12,13]. These systems benefit from the MW/AW assumption to the rotation estimation. They decouple the rotational and translational motion estimation and estimate drift-free rotational motion from structural regularities in man-made environments, which reduces the rotation error in the whole trajectory. However, the MW/AW assumption does not strictly hold in indoor scenes, which makes the range of applications limited. Zhou et al. [14] proposed using a single mean shift iteration algorithm to estimate the Manhattan dominant direction by a set of normal vectors. In [8], the absolute, drift-free rotation is estimated by tracking the MF from surface normal vectors. The translational motion is recovered by minimizing the de-rotated reprojection error with available depth point features. These approaches only use planes to search MF, which means that we at least need to detect two orthogonal planes in each frame. However, in practice, detecting two orthogonal planes is not very easy. To address this problem, Line and Plane based Visual Odometry (LPVO) [9] uses all tracked points (with and without depth) to estimate translation. They combine lines and planes to estimate drift-free rotation by a mean shift algorithm. To tackle the drift in translation estimation, Linear RGB-D SLAM (L-SLAM) [10] adds orthogonal planar features within a linear Kalman Filter framework based on LPVO. Atlanta Frame SLAM (AF-SLAM) [11] extends L-SLAM to cover more general structural environments with the AW assumption while maintaining linear computational complexity. [13] estimates the translation part by using point-line-plane tracking and adds parallel and perpendicular planar constraints to improve the tracking accuracy. [15] designed a short-term tracking module to track the clustered line features. In addition, a long-term searching module is designed to generate abundant sets of vanishing points (VPs) candidates and retrieve the optimal one. To optimize the model, [15] constructs a least square problem to provide refined VPs with the clusters of structural line features in each frame. To cope with dynamic scenarios, [16] uses a 2D tracker to track the moving object in bounding boxes. This method can effectively exclude the dynamic background and remove the outlier point and line features. [17] presents a semantic planar SLAM system to improve pose estimation and mapping by using cues from an instance planar segmentation network. [18] eliminates line features that are consistent with the motion direction. The structural line features are selected according to the direction information of vanishing points for a stronger geometric constraint on the pose estimation.

However, the decoupled scheme needs the MW assumption for every frame, which is very limiting. The indoor environments are not strictly conforming to the assumption, leading to performance degradation or even tracking failures. To address this issue, [19] uses planes to distinguish whether the scenes conform to the MW assumption, and then it chooses a decoupled or a non-decoupled tracking strategy to obtain the camera motion pose. Additionally, [5] proposes directly adding parallel and perpendicular constraints of planes to reduce drift errors in indoor environments without the MW assumption. [20] incorporates the MW assumption at the local map optimization stage instead of the tracking stage. Then, a local map optimization approach is proposed to combine the point and line reprojection error, the Manhattan Axes (MA) alignment, and the structural constraints of the scene. This method reduces the influence of punctual dissatisfaction with some constraints.

**Figure 1:**
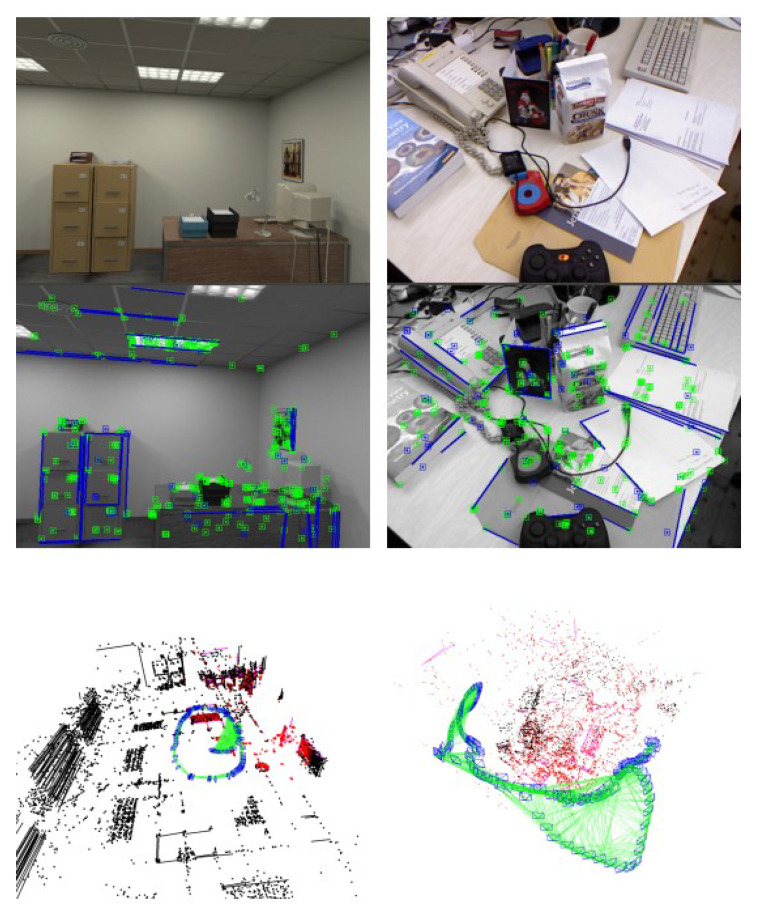
The proposed RGB-D VO system. **Top Left**: Structured scene. **Top Right**: Cluttered scene. **Bottom Left**: Sparse map in a structured scene. **Bottom Right**: Sparse map in a cluttered scene.

This paper proposes an RGB-D VO algorithm using points and lines to achieve robust pose features and good performance. We leverage the structural regularities in indoor scenes to improve tracking performance. The proposed method automatically recognizes whether the scene conforms to the MW assumption and chooses different tracking strategies. Moreover, we model the MW scenes as a Mixture of Manhattan Frames (MMF) [21], which consists of multiple independent MFs. We detect MFs with dominant directions extracted from parallel lines. Finally, we use dominant directions in local map bundle adjustment (BA) to improve rotation estimation. The proposed RGB-D VO system is shown in Figure 1. In summary, the main contributions of this work are as follows:

- A robust and general RGB-D VO framework for indoor environments is proposed. It is more suitable for real-world scenes because it can choose different tracking methods (decoupled and non-decoupled pose estimation methods) for different scenes.
- A novel drift-free rotation estimation approach is proposed. We detect the dominant directions for every frame by clustering the parallel lines. These dominant directions are tracked to detect MFs. Then, we use a mean-shift algorithm to obtain rotation estimation.
- An accurate and efficient local map bundle adjustment strategy combines points and lines reprojection errors with the rotation constraints from the multi-view dominant directions observations.

We compare the proposed method with other works in the literature, as shown in Table 1. All works are open source. To verify the effectiveness of the proposed method, we evaluate the proposed method on synthetic and real-world RGB-D benchmark datasets.

## 2. Materials and Methods

### 2.1. System Overview

**Figure 2:**
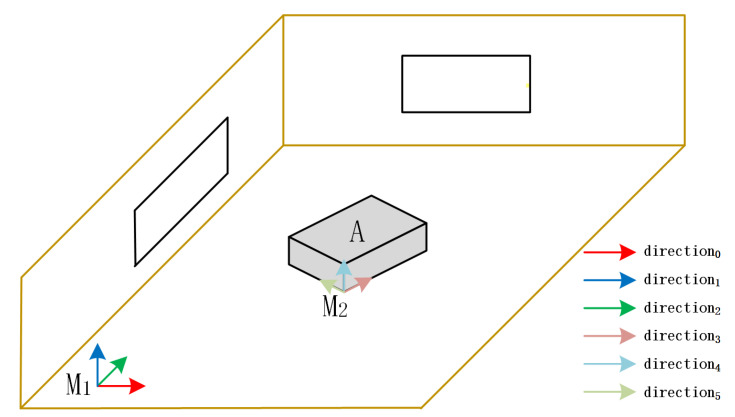
The dominant directions in the proposed method. The ${\mathrm{direction}}_{0}$ to ${\mathrm{direction}}_{5}$ constitute a set of unit vectors $\left\{d_{i}^{w}\right\}$. The ${\mathrm{direction}}_{0}$ to ${\mathrm{direction}}_{2}$ constitute the $M_{1}$.

In this work, we use $\left\{R_{kw},t_{kw}\right\}$ to represent the camera pose of the kth frame, where $R_{kw}\in SO\left(3\right)$ and $t_{kw}\in R^{3}$ denote the rotation and translation from the world frame to the camera frame, respectively. We also use a set of unit vectors $\left\{d_{i}^{w}\right\}$ to represent the dominant directions in the global map, and these vectors constitute all MFs saved in the Manhattan map G. Each MF contains the three mutually orthogonal dominant directions. These concepts are visualized in Figure 2. In addition, we use $\left\{d_{i}^{c_{k}}\right\}$ to represent the dominant directions in kth frame. The rotation matrix $R_{c_{k}m_{j}}\in SO\left(3\right)$ represents the orientation from jth MF to kth camera frame.

**Figure 3:**
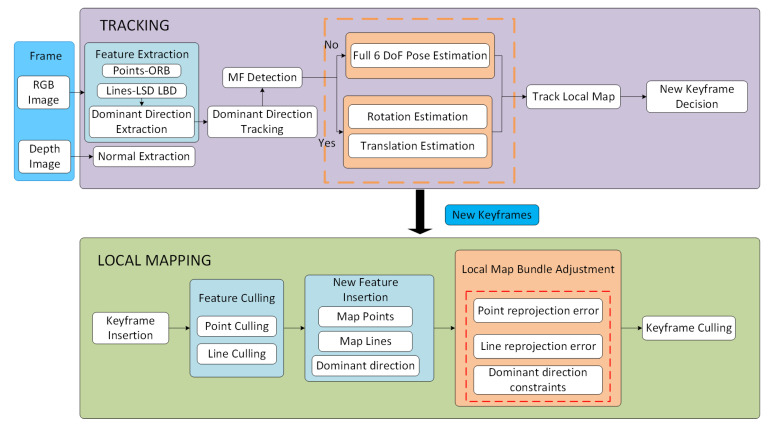
Overview of the proposed method.

With the RGB-D camera as the sensor input, the proposed system is built on top of the tracking and local mapping components of Oriented FAST and Rotated BRIEF SLAM2 (ORB-SLAM2) [22]. The overall framework is shown in Figure 3. We then describe each module of the proposed VO system.

The tracking thread is used to estimate the pose of each frame and select appropriate keyframes as input to the local mapping thread. In the tracking thread, for each frame, we extract point and line features from the RGB image and surface normals from the depth image, which are performed in parallel. Then, we extract the dominant directions from parallel lines to estimate the MFs in the current frame. The points, lines, and dominant directions are tracked and matched to estimate the camera pose. We divide the scenes into MW scenes and non-MW scenes. For MW scenes, we use a decoupled method to estimate the rotational and translational motion. For non-MW scenes, we combine point and line features with the dominant direction observations to estimate the whole 6 DoF camera pose. Based on the initial pose estimation, the camera motion is refined with the matched landmarks from the local map. Finally, the results on the keyframe are inferenced. We take both point and line features into account to decide whether a new keyframe should be inserted. Instead of a fixed reasonable threshold, the ratio-based method is use to create a new keyframe [20].

Map points, map lines, dominant directions, a set of keyframes, a covisibility graph, and a spanning tree jointly make up the stored map. The covisibility graph is maintained to link any two keyframes observing common landmarks. Whenever a keyframe is inserted, the local mapping thread is implemented to process the new keyframe and update the covisibility graph by the number of covisible landmarks. The map point culling and the map line culling are performed to improve tracking performance by retaining the high-quality map points and map lines. Furthermore, we merge the dominant directions to maintain the orientation difference between any two directions. Besides, a local map bundle adjustment procedure is performed to estimate keyframes poses, together with map points, map lines, and dominant directions observed by these keyframes. Finally, a keyframe culling procedure is conducted to remove the redundant keyframes. A keyframe is considered to be removed when more than 90% of map points can be observed by other keyframes (usually at least 3).

### 2.2. Feature Detection and Matching

In this paper, we use ORB features [23] to address the rotation, scale, and illumination changes. They can be extracted and matched quickly. The lines are extracted by Line Segment Detector (LSD) [24] and represented by Line Band Descriptor (LBD) [25]. The unit surface normal vectors are extracted from the depth image [9]. These procedures are conducted in parallel.

After extracting 2D features in the frame $F_{k}$, we use $p_{i}=\left(u_{i},v_{i}\right)$ to represent the 2D point feature and $l_{j}=\left(s_{j},e_{j}\right)$ to represent the line segment in image coordinates. Let $s_{j}\;\mathrm{and}\;e_{j}$ denote the start point and end point in the line segment $l_{j}$, respectively. The normalized line function of the observed 2D line segment is denoted as $l_{obs}={\left[\begin{array}{ccc} l_{1} & l_{2} & l_{3} \end{array}\right]}^{T}$, formally:

(1) $$l_{obs}=\frac{s_{j}\times e_{j}}{|s_{j}\left||e_{j}\right|}.\;$$

Once the 2D features have been detected and described, it is easy to obtain the 3D positions in camera coordinates according to the camera intrinsic parameters and the depth image. The 3D points and lines are denoted as $P_{i}^{c}$ and $L_{j}^{c}=\left(P_{j,start}^{c},P_{j,end}^{c}\right)$, respectively. To match point features, we still use the same strategy as ORB-SLAM2 to match. We jointly use both the LBD descriptor and geometric constraints to match line features between consecutive frames.

### 2.3. Dominant Direction

After obtaining the 3D position of lines, we classify the 3D line vectors to obtain parallel line clusters. The dominant directions are extracted from the parallel lines. The dominant directions are tracked and matched to detect the MFs and estimate the camera pose. We solve a least square problem for every parallel line cluster to determine its dominant direction:

(2) $$S^{T}d=0,\;$$

where $S=s_{i_{1\leq i\leq n}}\in {\mathbb{R}}^{3\times n}$ and n is the number of lines in this parallel line cluster. Each column $s_{j}$ represents a unit direction vector of the line in this cluster. Then, we obtain the initial set of dominant directions $\left\{d_{i}^{c_{k}}\right\}$ of the current frame $F_{k}$, and each dominant direction is a unit vector.

Unlike point and line features, the dominant directions are matched directly in the global map. To match the ith dominant direction $d_{i}^{c_{k}}$ of the kth frame and the jth dominant direction $d_{j}^{w}$ in the global map, we formulated it as:

(3) $$\cos\left(d_{i}^{c_{k}},d_{j}^{w}\right)=\left|\frac{d_{i}^{c_{k}}\cdot \left(R_{c_{k}w}d_{j}^{w}\right)}{|d_{i}^{c_{k}}\left||R_{c_{k}w}d_{j}^{w}\right|}\right|=\left|d_{i}^{c_{k}}\cdot \left(R_{c_{k}w}d_{j}^{w}\right)\right|\;$$

We choose those pairs $\left(d_{i}^{c_{k}},d_{j}^{w}\right)$ whose absolute values of cosine satisfy a given threshold (3° in this letter) as the candidate matches. As a result, we choose the dominant direction whose angular difference between $d_{i}^{c_{k}}$ and $d_{j}^{w}$ is the closest to 1 as the correct match.

Sometimes, the angular difference between two dominant directions in the global map may be smaller than the threshold after the local map BA. In that case, we merge the two dominant directions by an iterative to maintain the orientation difference between any two directions.

### 2.4. Manhattan Frame Detection

For MF $M_{i}$ in the kth frame, it can be represented by three mutually perpendicular dominant directions $\left\{d_{i,1}^{c_{k}},d_{i,2}^{c_{k}},d_{i,3}^{c_{k}}\right\}$. To detect an MF $M_{i}$ in $F_{k}$, we compute the angular difference between two different dominant directions in $\left\{d_{i}^{c_{k}}\right\}$. We think the two dominant directions are orthogonal if the angular difference meets the orthogonal threshold (at least 87° in this work). Any three dominant directions, which are mutually orthogonal, constitute an MF. If only two perpendicular dominant directions are found, the third direction can be obtained by taking the cross-product between the two dominant directions. At the same time, we add the newly created third dominant direction to the current frame’s dominant direction set $\left\{d_{i}^{c_{k}}\right\}$. The rotation matrix from this MF $M_{i}$ to the current frame is represented as $R_{c_{k}m_{i}}=\left\{d_{i,1}^{k},d_{i,2}^{k},d_{i,3}^{k}\right\}$.

Like the method in [19], we save the MFs in the scene to a Manhattan map G. Through the Manhattan map G, we can obtain the full and partial MF observations and the corresponding frames that observe the MF first.

### 2.5. Pose Estimation

Two different strategies are used to estimate the camera pose $T_{cw}=\left\{R_{cw},t_{cw}\right\}$ from world coordinates W to camera coordinates C, depending on whether the scenes conform to the MW assumption. For non-MW scenes, we directly estimate the 6 DoF camera pose with a feature tracking method. In MW scenes, we decouple the camera pose to separately estimate the rotational and translational motion.

#### 2.5.1. Non-MW Scenes

In non-MW scenes, the tracked dominant directions are used to estimate the camera motion by combining the point-line tracking. The dominant directions only provide the orientation constraints, independent of translation. Then, the full camera pose is estimated by minimizing the following cost function:

(4) $$\left\{R_{cw},t_{cw}\right\}=\underset{R,t}{arg\;min}\left[\underset{i\in \mathbb{P}}{\sum }\rho \left({\left\|e_{i}^{p}\right\|}^{2}\right)+\underset{j\in L}{\sum }\rho \left({\left\|e_{j}^{l}\right\|}^{2}\right)+\underset{k\in D}{\sum }\rho \left({\left\|e_{k}^{d}\right\|}^{2}\right)\right],$$

where ℙ, L, and D are the set of all point, line, and dominant direction matches, respectively. Let ρ denote the robust Huber cost function. The point reprojection error between observed 2D features and corresponding matched 3D features is defined as

(5) $$e_{i}^{p}=p_{i}-\pi \left(R_{cw}P_{i}^{w}+t_{cw}\right),$$

where $P_{i}^{w}\in {\mathbb{R}}^{3}$ is the 3D map point in world coordinates corresponding to the 2D point feature $p_{i}\in {\mathbb{R}}^{2}$ in the image plane. The projection function π transforms a 3D point $P^{c}$ in camera coordinates into the image plane:

(6) $$\pi \left(P^{c}\right)=\pi \left(\begin{array}{c} P_{x}\\ P_{y}\\ P_{z} \end{array}\right)=\left[\begin{array}{c} f_{x}\frac{P_{x}}{P_{z}}+c_{x}\\ f_{y}\frac{P_{y}}{P_{z}}+c_{y} \end{array}\right],$$

where the focal length $f_{x}$, $f_{y}$ and principal point $c_{x}$, $c_{y}$ belong to camera intrinsic parameters. The line reprojection error is formulated based on the point-to-line distance between the 2D line segment $l_{j}$ and the 3D endpoints $P_{j,start}^{w}$ and $P_{j,end}^{w}$ from the matched 3D line $L_{j}^{w}$. The error function is formulated as

(7) $$e_{j}^{l}=\left[l_{obs}^{T}\pi \left(R_{cw}P_{j,start}^{w}+t_{c}w\right),l_{obs}^{T}\pi \left(R_{cw}P_{j,end}^{w}+t_{c}w\right)\;\right].$$

We define the dominant direction observation errors based on the 3D–3D correspondence, formally:

(8) $$e_{k}^{d}=1-\left|\cos\left(\left(R_{cw}d_{k}^{w}\right)\cdot d_{k}^{c}\right)\right|$$

where $d_{k}^{w}$, $d_{k}^{c}$ are the dominant directions in world coordinates and camera coordinates, respectively. Then these data associations are employed to optimize the current camera pose using the Levenberg Marquardt (LM) algorithm implemented in g2o [26].

#### 2.5.2. MW Scenes

**Figure 4:**
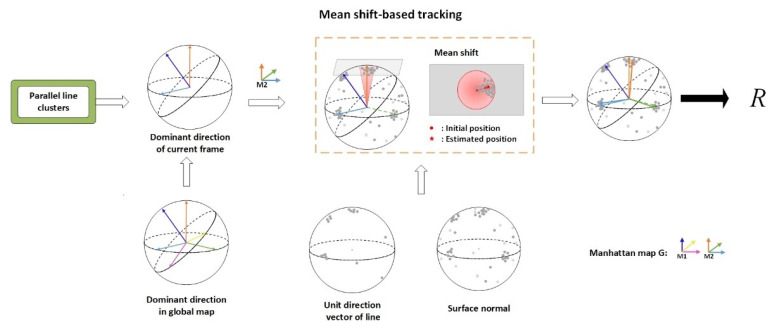
Rotation estimation in MW scenes. The proposed method first extracts the dominant directions from parallel lines and matches them in the global map. Secondly, we detect the MF $M_{2}$ by using dominant directions to obtain the initial rotation from MF to the current frame. The frame $F_{j}$ first observed this MF. Then, we use a mean shift-based tracking strategy to refine the rotation. Finally, we obtain the drift-free rotation using $F_{j}$ as the reference frame. The green dashed arrow indicates the virtual dominant direction created by the cross-product between the two extracted dominant directions.

Compared to estimating the camera pose directly from frame-to-frame tracking, the pose estimation can be decoupled in MW scenes. To reduce the drift caused by frame-to-frame tracking, we leverage the structural constraints in scenes to estimate the drift-free rotation. The translation estimation is recovered from the feature tracking. The process is shown in Figure 4.

For the rotation estimation, the set of dominant directions can be obtained using the method described in Section 2.3. Then, all MFs in the current frame can be detected using the method described in Section 2.4. To check whether an MF $M_{i}=\left\{d_{i,1}^{c_{k}},d_{i,2}^{c_{k}},d_{i,3}^{c_{k}}\right\}$ in the current frame $F_{k}$ is present in the Manhattan map G, we match the dominant direction in the current frame with the dominant direction in the global map using the method described in Section 2.3. For three dominant directions that constitute the MF $M_{i}$, if we can find that at least two directions are matched with the dominant directions in the global map and $M_{i}$ has been present in G, then we obtain the corresponding frame $F_{j}$ in which $M_{i}$ was first observed. If $F_{k}$ does not contain any previously observed MF, then we use the feature-tracking method (Section 2.5.1) instead of a decoupled method to solve the camera pose.

We use the popular mean shift algorithm [8,9,14] for MF tracking to estimate the rotation matrix. Firstly, we calculate the initial relative rotation $R_{c_{k}m_{i}}^{init}$ from MF $M_{i}$ to the current frame $F_{k}$ with the reference frame $F_{j}$ and the last frame $F_{l}$:

(9) $$R_{c_{k}m_{i}}^{init}=R_{c_{l}m_{i}}=R_{c_{l}w}R_{c_{j}w}^{T}R_{c_{j}m_{i}}.$$

Secondly, we transform the unit direction vectors of lines and the surface normal vectors in the current frame to MF $M_{i}$ using the transposed initial rotation matrix $R_{m_{i}c_{k}}^{init}$. We project the unit direction vectors of lines and the surface normal vectors onto tangent planes to compute a mean shift. Then, the mean shift result is transformed back to the unit sphere from the tangential plane. Finally, we obtain the updated rotation matrix $R_{c_{k}m_{i}}=\left[\begin{array}{ccc} r_{1} & r_{2} & r_{3} \end{array}\right]$. However, to make $R_{c_{k}m_{i}}$ still satisfy the orthogonality constraint, we transform $R_{c_{k}m_{i}}$ onto SO (3) manifold using singular value decomposition (SVD):

(10) $$R_{c_{k}m_{i}}=UDV^{T}=SVD\left(\left[\begin{array}{ccc} r_{1} & r_{2} & r_{3} \end{array}\right]\right),$$

(11) $${\hat{R}}_{c_{k}m_{i}}=UV^{T}.$$

Then, we can obtain the rotation matrix $R_{c_{k}w}$ from world coordinates to the current camera frame $F_{k}$ using the reference frame $F_{j}$:

(12) $$R_{c_{k}w}={\hat{R}}_{c_{k}m_{i}}R_{c_{j}m_{i}}^{T}R_{c_{j}w}.$$

More details on the sphere mean-shift method can be found in [8,9,14].

Once we obtain the drift-free rotation estimation, the 3 DoF translation estimation can be calculated by using the point-line reprojection errors. Note that we do not use the dominant direction observation errors in this process since they only provide rotational constraints. Furthermore, we simplify the original non-linear optimization problem into a linear one:

(13) $$t_{cw}=\underset{t}{arg\;min}\left[\underset{i\in \mathbb{P}}{\sum }\rho \left({\left\|{e_{i}^{\prime }}^{p}\right\|}^{2}\right)+\underset{j\in L}{\sum }\rho \left({\left\|{e_{j}^{\prime }}^{l}\right\|}^{2}\right)\right].$$

where ${e_{i}^{\prime }}^{p}$ and ${e_{j}^{\prime }}^{l}$ are the rotation-assisted point and line errors, respectively:

(14) $${e_{i}^{\prime }}^{p}=\left[\begin{array}{c} \left[{\left(R_{cw}P_{i}^{w}\right)}^{\left(3\right)}+t_{cw}^{\left(3\right)}\right]\frac{\left(u_{i}-c_{x}\right)}{f_{x}}-\left[{\left(R_{cw}P_{i}^{w}\right)}^{\left(1\right)}+t_{cw}^{\left(1\right)}\right]\\ \left[{\left(R_{cw}P_{i}^{w}\right)}^{\left(3\right)}+t_{cw}^{\left(3\right)}\right]\frac{\left(v_{i}-c_{y}\right)}{f_{y}}-\left[{\left(R_{cw}P_{i}^{w}\right)}^{\left(2\right)}+t_{cw}^{\left(2\right)}\right] \end{array}\right],$$

(15) $${e_{j}^{\prime }}^{l}=l_{1}f_{x}\left[{\left(R_{cw}P_{j,x}^{w}\right)}^{\left(1\right)}+t_{cw}^{\left(1\right)}\right]+l_{2}f_{y}\left[{\left(R_{cw}P_{j,x}^{w}\right)}^{\left(2\right)}+t_{cw}^{\left(2\right)}\right]+\left(l_{1}c_{x}+l_{2}c_{y}+l_{3}\right)\left[{\left(R_{cw}P_{j,x}^{w}\right)}^{\left(3\right)}+t_{cw}^{\left(3\right)}\right].$$

where we refer ${\left[\cdot \right]}^{\left(k\right)}$ as the kth row of a vector. $P_{j,x}^{w}$, $x=\left\{\;start,end\right\}\;$ represents the endpoints of the 3D line $\;L_{j}^{w}$. Then, we solve this BA problem using the LM algorithm.

After estimating the camera pose, we project the points, lines, and dominant directions in the local map to the current frame to obtain more correspondence. The current camera pose is optimized again with the resulting matches.

### 2.6. Local Map Bundle Adjustment

When a new keyframe K is inserted, the next step is to perform a local map BA procedure, which refines the camera poses and landmarks in the local map.

$\Gamma =\left\{P_{i}^{w},L_{j}^{w},d_{k},R_{l},t_{l}|i\in P,j\in L,k\in D,l\in K_{c}\right\}$ is the definition of the variable set to be optimized. $K_{c}$ represents all keyframes to be optimized, including the newly inserted keyframe and all local keyframes that are connected to it in the covisibility graph. P, L, and D represent all the map points, map lines, and dominant directions observed by these keyframes, respectively. We also fix some keyframes that observe these points, lines, and dominant directions but do not belong to $K_{c}$, denoted by $K_{f}$. We minimize the following cost function to estimate Γ:

(16) $$\Gamma =\underset{\Gamma }{arg\;min}\left[\underset{K\in \left\{K_{c}\cup K_{f}\right\}}{\sum }\left(\underset{i\in P}{\sum }\rho \left({\left\|e_{i}^{p}\right\|}^{2}\right)+\underset{j\in L}{\sum }\rho \left({\left\|e_{j}^{l}\right\|}^{2}\right)+\underset{k\in D}{\sum }\rho \left({\left\|e_{k}^{d}\right\|}^{2}\right)\right)\right].$$

## 3. Results

To evaluate the performance of the proposed method, we conduct experiments in synthesized and real-world sequences. Additionally, we compare it with other state-of-the-art approaches. All the experiments have been performed on an Intel Core i5-10400 CPU @ 2.90 GHz/16 GB RAM, without GPU parallelization. Additionally, we disable the bundle adjustment and loop closure modules of ORB-SLAM2 and SP-SLAM to make a fair comparison.

ORB-SLAM2 [22] is a feature-point based RGB-D SLAM system, and our method is based on it. MSC-VO is an RGB-D VO system using point, line, MW constraints, and a non-decoupled pose estimation method. ManhattanSLAM is an RGB-D SLAM system using point, line, plane, MMF constraints, and decoupled pose estimation methods. RGB-D SLAM is a SLAM system using point, line, plane, MW constraints, and decoupled pose estimation methods. SP-SLAM is an RGB-D SLAM system using point, plane, and non-decoupled pose estimation method. This information is also shown in Table 1.

### 3.1. ICL-NUIM Dataset

Imperial College London and National University of Ireland Maynooth (ICL-NUIM) [27] dataset is a synthesized dataset containing two low-texture scenes with ground truth trajectories: living room and office, as shown on the left side of Figure 1. The scenes are rendered based on a rigid Manhattan World model. Furthermore, this dataset contains large structured areas and low-textured surfaces such as floors, walls, and ceilings.

**Figure 5:**
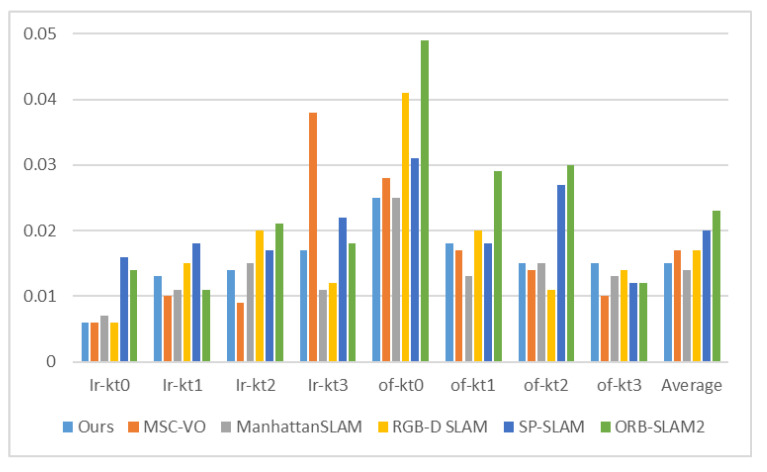
Comparison of ATE RMSE (M) for ICL-NUIM sequence.

**Figure 6:**
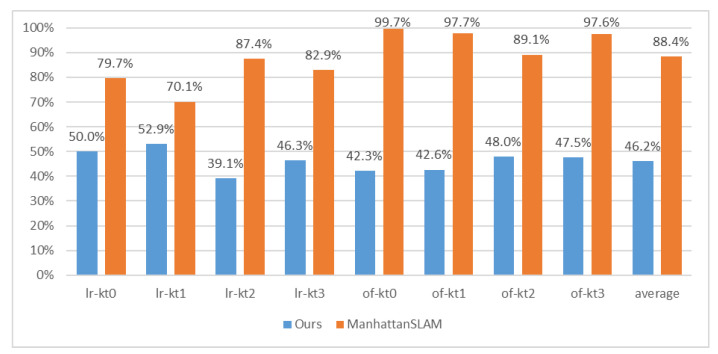
The percentage of MFs detected from each sequence in the ICL-NUIM dataset.

Table 2 shows the performance of our method based on the translation root mean square error (RMSE) of the absolute trajectory error (ATE). We compared the proposed method with the state-of-the-art systems, including MSC-VO, ManhattanSLAM, RGB-D SLAM, SP-SLAM, and ORB-SLAM2. The comparison of the RMSE is also shown in Figure 5. Figure 6 shows the percentage of MFs detected from each sequence in the ICL-NUIM dataset.

### 3.2. TUM RGB-D Dataset

**Figure 7:**
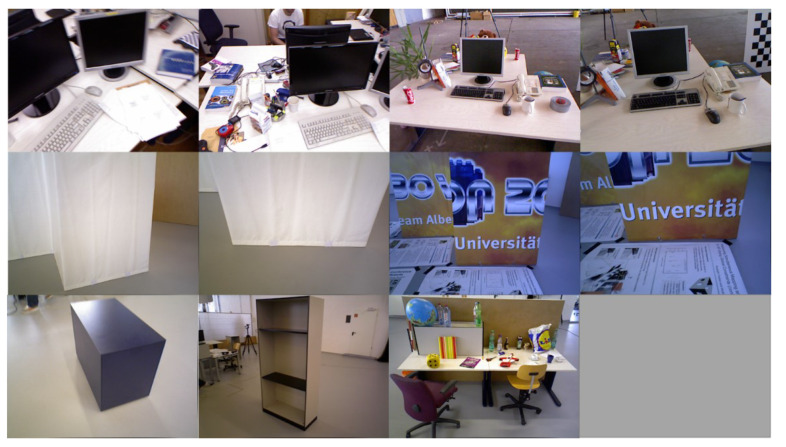
Sequences in TUM RGB-D dataset. The sorting is consistent with that in Table 3.

Technical University of Munich (TUM) RGB-D Benchmark [28] is a popular dataset to evaluate RGB-D VO/SLAM systems. Unlike the ICL-NUIM dataset, it consists of several real-world camera sequences, which contain different indoor scenes such as cluttered scenes, and different structure and texture scenes, as shown in Figure 7. Based on this, it can evaluate our system’s robustness and accuracy in both MW and non-MW scenes.

We selected 11 sequences in the TUM RGB-D dataset and divided them into three groups. Then we distinguished them according to the number of textures, structures and planes and whether they strictly follow the MW assumption. Table 3 shows the differences between sequences.

**Figure 8:**
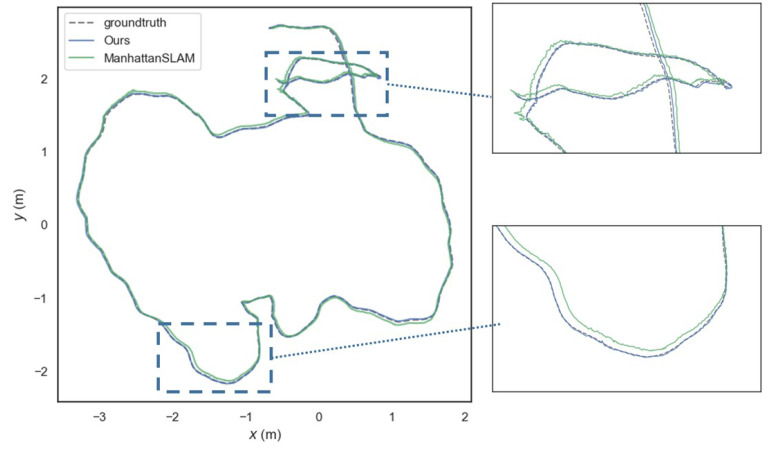
**Left**: Local map for the fr3-longoffice sequence. **Right**: Estimated trajectories with our method (blue) and ManhattanSLAM (green), and the ground truth (dashed grey) in TUM RGB-D dataset fr3-longoffice sequence.

**Figure 9:**
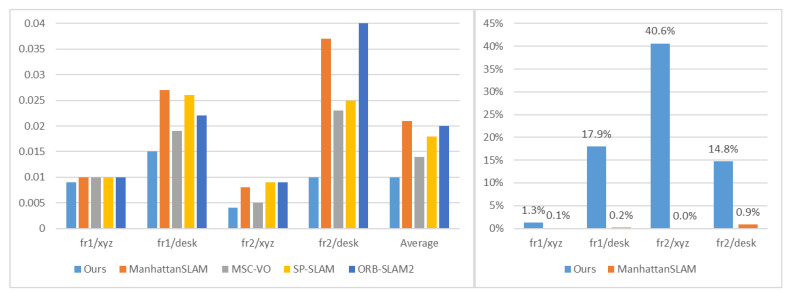
**Left**: Comparison of ATE RMSE (M) for sequence fr1/xyz, fr1/desk, fr2/xyz, fr2/desk. **Right**: The percentage of MFs detected from each sequence.

**Figure 10:**
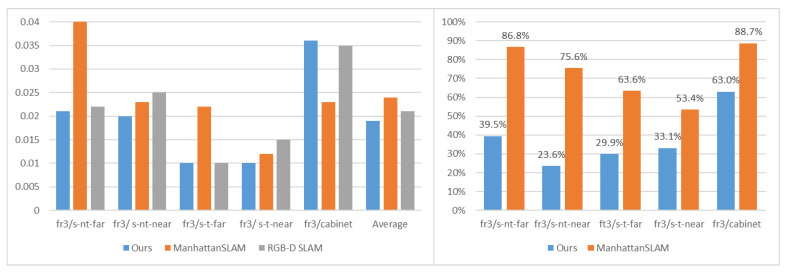
**Left**: Comparison of ATE RMSE (M) for sequence fr3/s-nt-far, fr3/s-nt-near, fr3/s-t-far, fr3/s-t-near, fr3/cabinet. **Right**: The percentage of MFs detected from each sequence.

**Figure 11:**
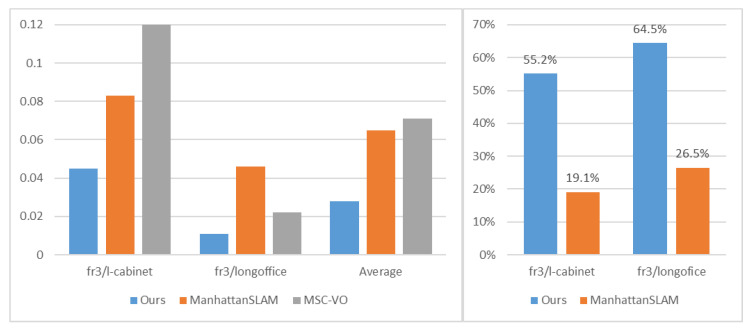
**Left**: Comparison of ATE RMSE (M) for sequence fr3/l-cabinet, fr3/longoffice. **Right**: The percentage of MFs detected from each sequence.

Table 4 shows the performance comparison of our method based on the translation RMSE (ATE), and other systems, including MSC-VO, ManhattanSLAM, RGB-D SLAM, SP-SLAM, and ORB-SLAM2. Local map for the fr3-longoffice sequence is shown in Figure 8. Relevant data are shown in Figure 9, Figure 10 and Figure 11.

### 3.3. Time Consumption

The average running time of each operation of the proposed method and ManhattanSLAM can be found in Table 5. We obtained the average results by running on seven different sequences in the TUM RGB-D benchmark.

### 3.4. Drift

**Figure 12:**
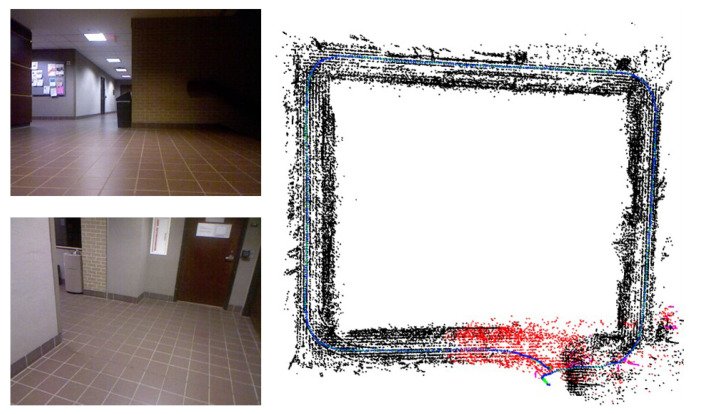
Drift for TAMU-RGB-D Corridor-A sequence.

We evaluated our system on the Texas A&M University (TAMU) RGB-D dataset [29] to test the amount of accumulated drift and robustness over time. Unlike the ICL-NUIM and TUM RGB-D datasets, the TAMU dataset does not provide ground-truth poses and contains long indoor sequences. Due to the camera trajectory being a loop, we can calculate the Trajectory Endpoint Drift (TED) [29], which computes the Euclidean distance between the starting and end points of the trajectory, to represent the accumulated drift. The output trajectory is shown on the right side of Figure 12.

## 4. Discussion

### 4.1. Localization Accuracy

#### 4.1.1. ICL-NUIM Dataset

The results are shown in Figure 5 and Table 2. Since there are rich structural regularities (enough lines and planes) and the highly present MW assumption in ICL-NUIM dataset, these are beneficial to the MW-based approaches. ManhattanSLAM shows the best quantitative results on average. Our method shows the second-best quantitative results on average, with a difference of 0.001 m. MSC-VO combines the structural constraints and MA alignment with the point line reprojection errors to optimize camera poses and shows the best quantitative results in four sequences. However, in sequence lr-kt3, it contains a perspective very close to the wall, which highly affects the MW detection, leading to the performance degradation of MSC-VO. Our method and ManhattanSLAM are more robust, as they can switch tracking strategies and adaptively estimate the camera motion.

Figure 6 shows the percentage of MFs detected from each sequence in the ICL-NUIM dataset. In ICL-NUIM dataset, it contains large structured areas. Since ManhattanSLAM uses plane features, it can detect MFs on 88% of all frames in sequence. The number of our method is 42%. However, it also leads to a 23 ms increase in time consumption. However, the average accuracy is only 0.001 m (6.6%) different. Time consumption data is described in Section 3.3.

#### 4.1.2. TUM RGB-D Dataset

The results are shown in Table 4. In the TUM RGB-D dataset, our method shows the best quantitative results. Only our method and ManhattanSLAM can obtain results in all sequences.

As shown in Table 4, in fr1 and fr2 sequences, the environments are cluttered and can be detected with few or no MFs using planes, which makes RGB-D SLAM, using a decoupled pose estimation method, track failure. ManhattanSLAM can robustly estimate a pose in these scenes by switching it to a feature-tracking method and performing an equivalent result to feature-based ORB-SLAM2 and SP-SLAM. However, the scenes also have a few structural characteristics such as lines, which makes our method achieve higher accuracy by using the dominant directions extracted from parallel lines.

For the fr3 sequence, the scenes contain different degrees of structure and texture. The proposed method can obtain the highest performance in six of seven except for cabinet. Only a few textures existed in four of seven sequences—the point-based method, ORB-SLAM2, is not able to find enough corresponding points, which results in tracking failure. As shown in Figure 8, after the camera runs a loop, the trajectory of our method does not drift significantly and achieves higher accuracy compared to other methods.

Next, we will further discuss the reason why our method is more accurate than ManhattanSLAM on the TUM RGB-D dataset. Relevant data are shown in Figure 9, Figure 10 and Figure 11.

The sequences of group 1 record a typical office scene, including desks, a computer monitor, a keyboard, a telephone, chairs, etc. The environments are cluttered and can be detected with few or no MFs using planes, as shown in Figure 9, less than 1%. Our method can still extract few structural characteristics such as lines, which means our method can achieve higher accuracy by using the dominant directions extracted from parallel lines.

The sequences of group 2 consist of multiple planes and can detect large MFs using planes, as shown in Figure 10. Our method can achieve higher accuracy. Although ManhattanSLAM can extract enough MFs, the planes in the first four sequences do not strictly follow the parallel or orthogonal relationship, and the forced use of the MW assumption will introduce redundant errors. Our method filters out non-orthogonal lines by line direction, making the real situation consistent with the assumption.

As shown in Figure 11, sequence fr3/l-cabinet contains some planes, but ManhattanSLAM does not extract enough MFs. With these sequences containing much texture and structure, our method can extract enough MFs, which makes our method achieve higher accuracy.

### 4.2. Time Consumption

Although the extraction of lines and surface normals is time-consuming for the pro-posed method, using multiple threads reduces the overall system time consumption, and we only need an average of 24.39 ms for the feature extraction. The local map BA procedure takes 183.34 ms on average, but it runs in a parallel thread. The whole tracking thread works at around 25 Hz. ManhattanSLAM takes 40 ms for superpixel extraction and surfel fusion and 67 ms for tracking on average. The whole tracking thread works at around 15 Hz.

The proposed method can work in real time. Our time consumption has decreased by 36%, and the accuracy has been maintained at the same level or beyond.

### 4.3. Drift

We employ Corridor-A and Entry-Hall sequences to evaluate the final trajectory drift. This dataset contains noisy depth data and low-texture floors and walls, as shown on the left side of Figure 12, which highly affect the camera pose estimation. As shown in Table 6, ManhattanSLAM achieved the best estimation results by adding plane features in the tracking process. The improvements of our method over the whole trajectory lengths of Corridor-A and Entry-Hall are 74.4% and 65.8%, respectively, compared to ORB-SLAM2. Compared with MSC-VO, which also uses point and line features, the improvements of our method are 12.1% and 29.0%.

## 5. Conclusions

In this letter, we propose an accurate and efficient RGB-D Visual Odometry system leveraging the structural regularity in indoor environments, which can robustly run in general indoor scenes. This is achieved by leveraging the dominant directions extracted from parallel lines in scenes to improve localization accuracy. On the one hand, the dominant directions can be used to solve the drift-free rotation estimation in MW scenes. On the other hand, they can also provide a rotation constraint to incorporate point and lines reprojection errors to optimize the camera pose. All these contributions can improve the accuracy of the computed trajectory for our method, as shown in our experiments. Furthermore, our pipeline is designed to address the different scenes: MW scenes and non-MW scenes, which means our system can work in a wider range of environments.

The estimation accuracy of the line affects the calculation of the dominant direction. If the uncertainty of the 3D coordinates of the recovered line is too large, the calculation and matching of the dominant direction will be affected, and the relative MF cannot be matched. In the future, we would like to add a loop closure module and improve the dominant direction detection to further discard unstable observations. We will also try to implement the proposed method with a monocular camera and IMU, which is beneficial for the Manhattan Frame detection, and possibly extend it to outdoor environments.

## Figures and Tables

**Table 1:** Comparison of the proposed method with other works in the literature.

Method	Year	Feature Types	Assumption	Pose Estimation Method
Ours	2022	Point, Line, direction	MMF	decoupled
MSC-VO	2021	Point, Line	MW	non-decoupled
ManhattanSLAM	2021	Point, Line, Plane	MMF	decoupled
RGB-D SLAM	2021	Point, Line, Plane	MW	decoupled
SP-SLAM	2019	Point, Plane	×	non-decoupled
ORB-SLAM2	2017	Point	×	non-decoupled

× represents no assumption.

**Table 2:** Comparison of ATE RMSE (M) for ICL-NUIM sequence.

Sequence	Ours	MSC-VO	ManhattanSLAM	RGB-D SLAM	SP-SLAM	ORB-SLAM2
Ir-kt0	**0.006**	**0.006**	0.007	**0.006**	0.016	0.014
Ir-kt1	0.013	**0.010**	0.011	0.015	0.018	0.011
Ir-kt2	0.014	**0.009**	0.015	0.020	0.017	0.021
Ir-kt3	0.017	0.038	**0.011**	0.012	0.022	0.018
of-kt0	**0.025**	0.028	**0.025**	0.041	0.031	0.049
of-kt1	0.018	0.017	**0.013**	0.020	0.018	0.029
of-kt2	0.015	0.014	0.015	**0.011**	0.027	0.030
of-kt3	0.015	**0.010**	0.013	0.014	0.012	0.012
Average	0.015	0.017	**0.014**	0.017	0.020	0.023

The best result for each sequence is shown in bold.

**Table 3:** Differences between sequences in TUM RGB-D dataset.

Group	Sequence	Texture	Structure	Plane	Strict Follow the MW Assumption
1	fr1/xyz	high	middle	low	middle
	fr1/desk				
	fr2/xyz				
	fr2/desk				
2	fr3/s-nt-far	low	high	high	low
	fr3/s-nt-near				
	fr3/s-t-far	high			
	fr3/s-t-near				
	fr3/cabinet	low			high
3	fr3/l-cabinet	high	middle	middle	middle
	fr3/longoffice				

**Table 4:** Comparison of ATE RMSE (M) for TUM RGB-D sequence.

Group	Sequence	Ours	MSC-VO	ManhattanSLAM	RGB-D SLAM	SP-SLAM	ORB-SLAM2
1	fr1/xyz	**0.009**	0.010	0.010	×	0.010	0.010
	fr1/desk	**0.015**	0.019	0.027	×	0.026	0.022
	fr2/xyz	**0.004**	0.005	0.008	×	0.009	0.009
	fr2/desk	**0.010**	0.023	0.037	×	0.025	0.040
	Average	**0.010**	0.014	0.021	*	0.018	0.020
2	fr3/s-nt-far	**0.021**	0.077	0.040	0.022	0.031	×
	fr3/s-nt-near	**0.020**	×	0.023	0.025	0.024	×
	fr3/s-t-far	**0.010**	-	0.022	**0.010**	0.016	0.011
	fr3/s-t-near	**0.010**	-	0.012	0.015	0.010	0.011
	fr3/cabinet	0.036	-	**0.023**	0.035	×	×
	Average	**0.019**	*	0.024	0.021	*	*
3	fr3/l-cabinet	**0.045**	0.120	0.083	0.071	0.074	×
	fr3/longoffice	**0.011**	0.022	0.046	-	-	0.021
	Average	**0.028**	0.071	0.065	*	*	*

× represents tracking failure—means result is not available. * represents that at least one sequence tracking failure or not available. The best result for each sequence is shown in bold.

**Table 5:** Mean execution time (TUM RGB-D benchmark).

Method	Tracking			Local Mapping
Ours	Feature Extrac.	Pose Estim.	Total (Hz)	Local Map BA
	24.39	12.59	25	183.34
ManhattanSLAM	superpixel extraction and surfel fusion		Total (Hz)	-
	37.8		16	-

**Table 6:** Comparison of the accumulated drift (m) in TAMU RGB-D sequence.

Sequence	Ours	MSC-VO	ManhattanSLAM	ORB-SLAM2	Length (m)
Corridor-A	0.80	0.91	0.53	3.13	82
Entry-Hall	0.76	1.07	0.39	2.22	54
